# Thermal Expansion and Thermal Conductivity of Ni/Graphene Composite: Molecular Dynamics Simulation

**DOI:** 10.3390/ma16103747

**Published:** 2023-05-15

**Authors:** Ramil T. Murzaev, Karina A. Krylova, Julia A. Baimova

**Affiliations:** Institute for Metals Superplasticity Problems of the Russian Academy of Sciences, Ufa 450001, Russia; mur611@mail.ru (R.T.M.); bukreevakarina@gmail.com (K.A.K.)

**Keywords:** crumpled graphene, Ni/graphene composite, molecular dynamics, thermal conductivity, thermal expansion coefficient

## Abstract

In the present work, the thermal conductivity and thermal expansion coefficients of a new morphology of Ni/graphene composites are studied by molecular dynamics. The matrix of the considered composite is crumpled graphene, which is composed of crumpled graphene flakes of 2–4 nm size connected by van der Waals force. Pores of the crumpled graphene matrix were filled with small Ni nanoparticles. Three composite structures with different sizes of Ni nanoparticles (or different Ni content—8, 16, and 24 at.% Ni) were considered. The thermal conductivity of Ni/graphene composite was associated with the formation of a crumpled graphene structure (with a high density of wrinkles) during the composite fabrication and with the formation of a contact boundary between the Ni and graphene network. It was found that, the greater the Ni content in the composite, the higher the thermal conductivity. For example, at 300 K, λ = 40 W/(mK) for 8 at.% Ni, λ = 50 W/(mK) for 16 at.% Ni, and λ = 60 W/(mK) for 24 at.% Ni. However, it was shown that thermal conductivity slightly depends on the temperature in a range between 100 and 600 K. The increase in the thermal expansion coefficient from 5 × 10${}^{-6}$ K${}^{-1}$, with an increase in the Ni content, to 8 × 10${}^{-6}$ K${}^{-1}$ is explained by the fact that pure Ni has high thermal conductivity. The results obtained on thermal properties combined with the high mechanical properties of Ni/graphene composites allow us to predict its application for the fabrication of new flexible electronics, supercapacitors, and Li-ion batteries.

## 1. Introduction

Nowadays, the demand for new materials with unique mechanical and physical properties is constantly growing. The search for graphene/metal composites is a new and actively developing branch of composite technologies [1,2,3,4]. The main component of these composites is graphene, which has low weight, high strength, and flexibility, as well as high electrical and thermal conductivity [5,6,7,8,9]. Such unique properties of graphene, added to the metal matrix, lead to the formation of composites with special properties that have use for future applications.

To date, the mechanical properties of metal matrix composites reinforced by graphene have been widely studied [10,11,12,13,14,15]. Commonly, a metal matrix (for example aluminum, copper, or nickel) is reinforced by graphene nanoflakes/nanoribbons or carbon nanotubes (CNTs). Both carbon polymorphs can considerably change the properties of metal and vise-versa [16,17,18]. It was shown that the Fe doping of graphene considerably affects its electronic, magnetic, and optical properties [16]. For example, graphene nanosheets in the aluminum matrix, added during stir casting, have increased hardness and strength by 24.65% and 23.61%, respectively, compared with pure aluminum [14]. Graphene-reinforced Al matrix composites obtained by extrusion also showed improved mechanical properties [11,12,19,20]. The corrosion properties and structural homogeneity of the aluminum alloy surface coatings increased through graphene reinforcement [1,21]. A Cu matrix filled with graphene nanoparticles also has increased strength, as well as improved electrical and thermal conductivity [10,15,22]. It has been experimentally shown that a graphene-reinforced nickel matrix composite resulted in higher strength, while its plasticity remained unchanged [13]. The yield strength and ultimate tensile strength of such a composite were much higher than that of pure Ni. It has been shown that the coating of a Ni-rich oxide with graphene provides high specific capacity, at 5.4 mAh cm${}^{-2}$ (increased by 38%), and high volumetric capacity, at 863 mAh cm${}^{-3}$ (increased by 34%) at 1.1 mA cm${}^{-2}$ [23]. These new composite materials can be used to improve cathodes in lithium-ion batteries, which may increase their capacity while maintaining the weight of the product.

In addition to high strength, graphene shows unique thermal properties—these include high thermal conductivity and a negative thermal expansion coefficient (TCC and TEC, respectively). It has been experimentally shown that the thermal conductivity of single-layer graphene at room temperature is in the range from 4840 W/(K m) to 5300 W/(K m) [24,25,26]. This value exceeds the thermal conductivity for diamonds (1000–2000 W/(K m) [27]), graphite (2000–6000 W/(K m)) [28,29]), and carbon nanotubes (3000–3500 W/(K m) [30,31]) at room temperature. The wide difference range for the thermal conductivity of carbon structures is associated with a number of different factors: experimental or simulation technique, temperature, the purity of the materials, and the lack of defects [32]. The size of graphene also strongly affects its thermal conductivity: graphene with a larger size has higher thermal conductivity [33]. With the temperature increase, the thermal conductivity of graphene decreases, as well as for the other carbon structures [26,28,33].

A negative TEC for graphene has been observed in the temperature range from 200 to 400 K [34,35,36]. This is explained by the superposition of two processes: (i) stretching of the bonds and (ii) the formation of ripples and folds on the graphene surface [36,37,38]. In [39], it was shown that the difference between the TEC of defect-free graphene in the armchair and zigzag directions was 9%, and the introduction of vacancy defects led to an increase in the negative TEC value.

Thus, together with the improvement of mechanical properties, graphene reinforcement results in the improvement of thermal properties. The study of such properties of graphene/metal composites is of high importance for the fabrication of new electronic devices such as chemical sensors, energy storage, catalysis, hydrogen storage, batteries, and supercapacitors [3,4,40]. The application of composites based on graphene aerogel for Li-ion batteries, flow batteries, and supercapacitors was shown in [3]. Moreover, considerable progress in the application of graphene-based composites toward energetic materials has been shown recently in [4]. In [41], it was shown that the boundary thermal conductivity of an Al/graphene composite decreased with an increase in the number of graphene layers placed between aluminum single crystals. Furthermore, the total thermal conductivity of the Al/graphene composite increased with an increase in the graphene content. Aluminum and Al–Cu composites reinforced with diamond nanoparticles also have a high thermal conductivity of 670 and 330 W/(m K), respectively, [42,43]. In the experimental work of [2], a metal matrix graphene/Ni/Cu multilayer composite with high thermal conductivity (1042.4 W/(m K)) was obtained by electrodeposition, which was followed by vacuum hot pressing. Studies of the boundary thermal conductivity of Cu/graphene and Ni/graphene composites showed that the introduction of one layer of graphene between metal single crystals led to a significant increase in thermal conductivity. The addition of even a small amount of graphene to the Cu matrix led to a TEC decrease. In [44], a Cu/graphene composite, obtained by dispersion electroplating, showed a decrease in the TEC by 30% compared to that of pure Cu.

One of the interesting graphene/metal composite morphologies is a graphene network filled with metal nanoparticles. Such graphene networks, also called graphene aerogel/fiber/crumpled graphene or 3D graphene, exhibit very different morphologies with random porous structures and a pore size from tens to hundreds of nm [45]. For some morphologies, graphene flakes can be stacked to form an ordered porous structure or be separated and interconnected by van der Waals interaction. Such structures can be composed of single-layer graphene or few-layer graphene flakes. The construction of such graphene networks in the metal matrix has been intensively studied over the last few years [46,47,48,49]. There is a wide variety of graphene/metal composite morphologies. For example, one of the first considered structures was a metal matrix with a graphene layer on the inside to reinforce the metal structure. Such composites have one important disadvantage—high anisotropy. The composite strength is increased when tension is applied along the graphene plane, but this strength is much lower when tension is applied normally to the graphene layer. In recent years, the following new morphology became much more popular—a graphene network filled with metal [47,50,51,52]. It is much easier to fabricate, since metal nanoparticles can be a catalyst, and such structures have much higher strength due to the high strength of the graphene network itself.

Such metals as Cu, Ni, Ag, and Au are of great interest from this point of view, and, in the present work, Ni was chosen as the filler for crumpled graphene. It was shown that Ni nanoparticles grow densely and uniformly inside graphene aerogel [46]. For example, such Ni/graphene composites have good mechanical properties [48], and are potentially very promising in electronic and energy applications. However, the flake morphology affects the plastic activity and the hardness of the composites and can weaken the structure [53]. Plenty of work has been devoted to the study of the synthesis of such composites and to their electronic and mechanical properties, while thermal properties are almost not studied.

A promising method for estimating the phonon thermal conductivity of such composites is nonequilibrium molecular dynamics (MD). There are several algorithms for calculating the thermal conductivity using the LAMMPS (large-scale atomic/molecular massively parallel simulator) [54,55] package, all of which accurately predict thermal properties at temperatures from 100 K and above when the contribution of electronic thermal conductivity not significant. Note that strict first-principle calculations for evaluating the thermal properties of materials are limited to the zero temperature [56].

In the presented work, the thermal conductivity and TEC of graphene/Ni composites were studied in a wide temperature range by MD simulation. The methodology for the calculation of thermal properties of graphene/metal composites was developed and tested on the graphene/Ni composite. The proposed methodology will further allow for calculations of the thermal properties of such complex structures as graphene networks filled with other atomic clusters. Both metallic fillers and other elements (such as H, O, or N atoms) can be added to the graphene network. This methodology can be applied to calculate the thermal conductivity of crumpled graphene itself, since this structure is proposed for new supercapacitors [3,4,40], which can be considerably affected by thermal properties. The obtained thermal characteristics also allow us to understand how the ratio of metal filler affects the thermal properties of the graphene network.

## 2. Simulation Details

### 2.1. Composite Structure

**Figure 1:**
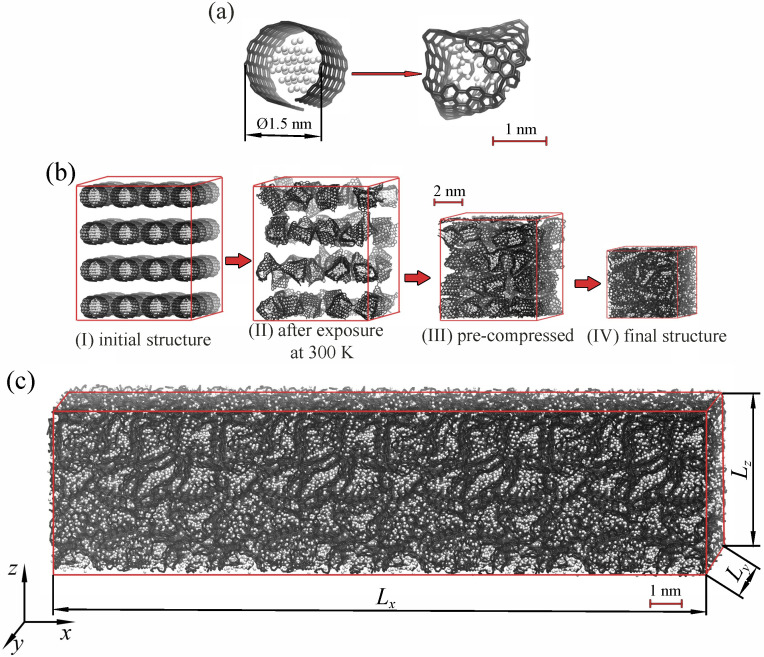
(**a**) Structural element of the composite in the initial state and after exposure at 300 K. (**b**) The schematic of fabrication of a Ni/graphene composite: (**I**) initial structure, (**II**) after exposure at 300 K, (**III**) after pre-compression at 300 K, and (**IV**) after high-temperature hydrostatic compression. (**c**) The large composite structure for the calculation of thermal conductivity. Ni atoms are shown in white, and carbon atoms are shown in black.

In Figure 1a, a structural element of the composite—a graphene flake (GF) with the Ni nanoparticle inside—is shown in the initial state and after relaxation. Here, Ni was chosen as the filler for the graphene network, since this metal has a very high adhesion to graphene. It is often used for graphene fabrication, and Ni nanoparticles can be catalysts for CNT or graphene growth [57,58]. Graphene flakes in similar experimentally observed graphene architectures can be very different [45]. However, for simplicity, in the present work, one special morphology was considered—a graphene network composed of single-layer graphene flakes interconnected by van der Waals interactions. The edge atoms of the graphene flake have sp hybridization, which makes it easy to form new bonds with neighboring flakes. The modification of graphene edges by hydrogen atoms was not considered for the sake of simplicity. The size of the graphene flakes was about 3–4 nm along both directions.

The structural element was randomly rotated and translated along the *x*-, *y*-, and *z*-axis to obtain the three-dimensional (3D) composite precursor, which was then exposed to 300 K temperatures for 20 ps to obtain a more realistic structure with crumpled graphene flakes. After that, hydrostatic compression at temperatures of 300 K was applied to remove empty space from the structure. It should be noted that, during this step, new covalent bonds are not formed between neighboring GFs, since higher temperatures are required [59]. Finally, the composite was obtained by hydrostatic compression at temperatures of 1000 K. This fabrication technique was developed previously for Ni/graphene, Cu/graphene, and Al/graphene composites [48,50,60,61,62,63]. All the simulation steps are shown in Figure 1b—from the initial state to the fabricated composite. The compression temperature was chosen as 0.6–0.7 $T_{m}$ (where $T_{m}$ is the melting temperature of metal nanoparticles). For the Ni nanoparticle, the $T_{m}$ is 1723 K [60]. Note that the melting temperature of graphene is about 5000 K [59].

Three different composites with Ni content were considered—8, 16, and 24 at.% Ni. Each structural element consisted of 252 carbon atoms, while the sizes of the nanoparticles were different. For the composite with 8 at.% Ni, there were 21 Ni atoms in each graphene flake. Analogously, for the composite with 16 (24) at.% Ni, there were 47 (78) Ni atoms in each GF. The initial diameters of the Ni nanoparticles composed of 21, 47, and 78 atoms were *d* = 6.2, 9, and 10.4 Å, respectively.

The compressed structure was relaxed until thermodynamic equilibrium was reached. At first, the composite was equilibrated at NPT ensembles at each studied temperature in a range from 0 to 1500 K, with a step of 100 K for 1 ps. This was enough to obtain the simulation cell with zero pressure. Then, thermalization of the structure was conducted using the NVE ensemble for 1 ps to achieve the constant temperature. Thermalization was conducted at each studied temperature in a range from 0 to 1500 K with a heating rate of 3 K/ps. Note that the heating rate was chosen to provide the fluctuation processes. After that, the final size of the Ni/graphene composite was about 50 × 50 × 50 Å, with a density of 5.15 g/cm${}^{3}$. This size of the simulation cell was enough to calculate the thermal expansion coefficients.

To calculate the thermal conductivity of the composite, the initial size of 50 × 50 × 50 Å of the simulation is too small. Thus, the Ni/graphene composite with the 210 × 50 × 50 Å size was chosen. This new simulation cell was also equilibrated at NPT ensembles at each studied temperature in a range from 100 to 600 K, with the step of 100 K for 1 ps. Then, it was thermalized using the NVE ensemble for 1 ps. Thermalization was conducted at each studied temperature in a range from 100 to 600 K, with the step of 100 K.

Note that, in this work, the results obtained for a composite were compared with that for pure, single-crystal Ni. Thus, pure Ni fcc crystals with the sizes of 51 × 51 × 51 Å and 205 × 51 × 51 Å were considered. The *x*-, *y*-, and *z*-axis coincided with the [100], [010], and [001] crystallographic directions, respectively. The lattice parameter was 3.518 Å. The number of Ni atoms was 13,500 and 54,000 atoms, respectively. The equilibration and thermalization of Ni samples were carried out under the same conditions as for the composite.

The equations of motion for the atoms were integrated using the fourth-order Verlet method, with a time step of 0.2 fs. Periodic boundary conditions were applied along the *x*-, *y*-, and *z*-axis. For each state, three to five measurements of the TCC and TEC values were carried out. The standard deviation of these values ranged from about 5–7% at each temperature. To visualize the structure, the molecular graphics program in VMD (visual molecular dynamics) was used, which allows for the display and analysis of structural changes at certain simulation stages [64].

### 2.2. Interatomic Potentials

Three different interactions were described by interatomic potentials: Ni-Ni, C-C, and Ni-C. The embedded atom method (EAM) was used to describe the Ni-Ni interactions [65], and AIREBO potentials were adopted for C-C interactions [66]. Both potential functions are well-known and can be effectively used for the simulation of various properties of metallic [67,68] and carbon structures [69,70].

The Ni-C interaction was described using the Morse interatomic potential with the parameters obtained by ab initio calculations [71,72]: the well potential depth was $D_{e}$ = 0.433 eV, the equilibrium distance of the Ni-C bond was $R_{e}$ = 2.316 Å and the parameter controlling the “width” of the potential well was β = 3.244 1/Å. These potentials are well tested and have been used to simulate both the physical and mechanical properties of similar materials [63,70,73,74,75].

### 2.3. Calculation of Thermal Expansion Coefficient

After thermodynamic equilibrium at 0 K was reached, measurements of the TEC α values of the Ni/graphene composite and pure Ni (the size of the simulation cell was 51 × 51 × 51 Å) were conducted. The simulation cell was equilibrated at each temperature from 0 K to 1500 K with the step of 100 K and the heating rate of 3 K/ps. During the entire process, the size of the simulation cell was allowed to change anisotropically. As the temperature of the system increased, the size of the simulation cell along the *x*-, *y*-, and *z*-axis was recorded each 0.05 ps and used to calculate the thermal expansion of the system.

The TEC is calculated using the approaches from [29,76,77]:

(1) $$\alpha =\frac{1}{L_{0}}\frac{\Delta L}{\Delta T},$$

where $L_{0}$ is the initial equilibrium length of the simulation cell at 0 K, and ΔL/ΔT is the change in the length of the simulation cell with temperature $\Delta L=L_{final}-L_{0}$. To calculate *L*, the average size of the simulation cell along three dimensions was calculated as $L=(L_{x}+L_{y}+L_{z})/3$, where $L_{x}$, $L_{y}$, $L_{z}$ are the sizes of the simulation cell along the *x*, *y*, and *z*-axis, respectively, at every time step.

Thus, the TEC of the Ni/graphene composites was estimated from the relative elongations of the samples ($\Delta L/L_{0}$) in a wide temperature range.

### 2.4. Calculation of the Thermal Conductivity

The heat transfer in solid bodies can be carried out by both phonons (lattice vibrations) and by electrons [78,79,80,81,82]. However, for most metals, the electronic thermal transport at low temperatures (*T* < 100 K) is predominant. In this work, the thermal conductivity of the composites was estimated at temperatures above 100 K, in order to exclude electronic conductivity, which cannot be taken into account in an MD simulation.

**Figure 2:**
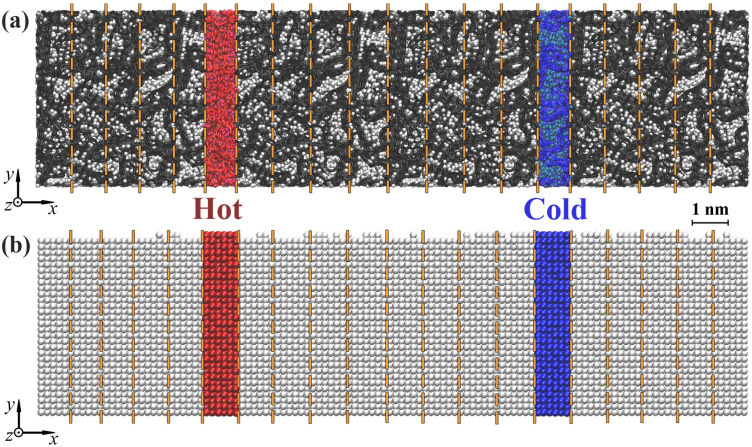
Molecular dynamics setups for evaluating the thermal conductivity of (**a**) Ni/graphene composite and (**b**) pure Ni. The red and blue regions show a “hot” and “cold” source. Dashed orange lines show the slabs. Ni atoms are shown in white, and carbon atoms are shown in black.

After the thermodynamic equilibrium of the system was reached, the computational cell was divided along the *x*-axis into 20 identical slabs of 10–10.5 Å in width. In Figure 2, the simulation cells of (a) Ni/graphene composite and (b) pure Ni are presented as the MD setups for thermal conductivity calculations. The heat source is the sixth (red) slab, and the sink is the fifteenth (blue) slab. Between the“hot” and “cold” slabs, a temperature gradient was set along the length direction, which resulted in a heat flux ΔQ. The temperatures of the “hot” and “cold” slabs are T+ΔT and T−ΔT, respectively. The value of ΔT was chosen as 10% of the system temperature. The temperature of the other slabs at the beginning of the simulation was 300 K.

During the simulation, the temperature in the system was controlled under a canonical NPT ensemble. The temperature distribution in each of the 20 layers was monitored throughout the simulation process.

The Fourier law is used to calculate the heat flow [83,84,85,86,87]:

(2) $$\frac{\Delta Q}{\Delta \tau }=-\lambda \frac{\Delta T}{\Delta x}S,$$

where ΔQ/Δτ is the transferred thermal energy from the hot and cold regions during the time range within which the transferred energies are calculated, *S* is the cross–sectional area, and ΔT/Δx is the temperature gradient. Thus, the thermal conductivity λ is calculated as:

(3) $$\lambda =-\frac{\Delta Q}{\Delta \tau }\frac{\Delta x}{\Delta T}\frac{1}{S}.$$

The described method for estimating the heat flux occurring between “hot” and “cold” regions was proposed in [83], which was later improved in [84], and it was implemented using LAMMPS algorithms. As a result, it was shown that this algorithm for NEMD (non-equilibrium molecular dynamics method) simulations of thermal gradients performs a simulation of a stable heat flow, which makes it possible to estimate the thermal conductivity of the system under study with sufficiently high accuracy. Subsequently, this method has been used to estimate the TCC values of various metal and carbon systems [41,86,88,89,90].

## 3. Results

### 3.1. Thermal Expansion Coefficient

**Figure 3:**
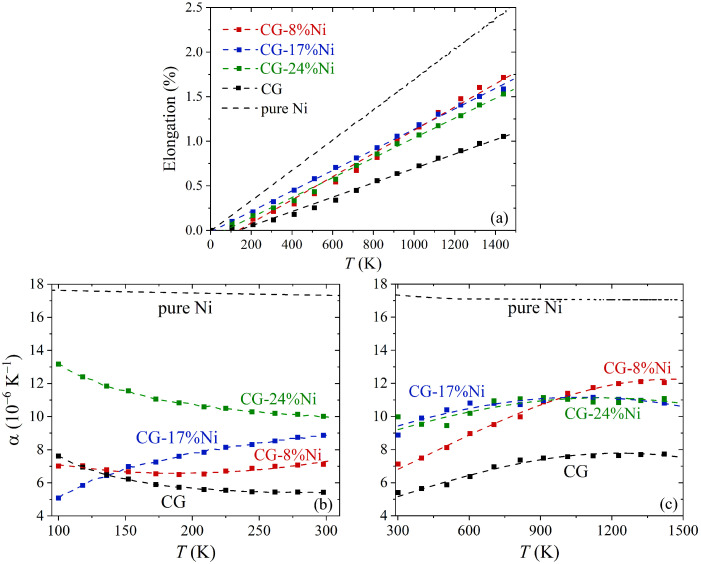
The relative elongation (**a**) and the temperature expansion coefficient (α) (**b**,**c**) of the Ni/graphene composite, crumpled graphene (CG), and pure Ni as a function of temperature.

The relative elongation $\Delta L/L_{0}$ as a function of temperature is shown in Figure 3a for all the studied composite materials compared with pure Ni and crumpled graphene. For all the structures, the relative elongation of the samples linearly increased with temperature in a whole temperature range. The same behavior was shown for Ni in the experiment, and the MD simulation [91,92] featured linear expansion in the temperature range from 0 to 1800 K.

Crumpled graphene showed the lowest ratio of linear expansion. There were no direct observations of thermal expansion for the 3D crumpled graphene, but some results were obtained for one crumpled/rippled graphene layer [93]. It has been shown that crumpled graphene sheets can exhibit a wide range of coefficients of thermal expansion from large negative to large positive by controlling the folding pattern. In our work, we had a considerably compressed crumpled structure, which was composed of a number of graphene flakes. Thus, it cannot be directly compared to the literature.

The presence of Ni in the pores of crumpled graphene led to an increase in the relative elongation of the samples compared to crumpled graphene (see Figure 3a).

The TEC as the function of time is shown in Figure 3 for the temperature range 100–300 K (b) and for 300–1400 K (c). It can be seen that the crumpled graphene had the lowest α compared to the composites and Ni. In [93], it was shown that, for one crumpled graphene layer, controlling ripples can result in achieving a wide range of the TEC from positive to negative. In our work, the TEC for crumpled graphene considerably changed at room temperature and changed only slightly at *T* > 300 K. It should be mentioned that, for our structure, the crumpled graphene was highly compressed, and the ripples had rigid corners, which are very different results from previously studied structures. The minimum TEC value of the crumpled graphene was observed at 300 K and equal to 5.4 × 10${}^{-6}$ K${}^{-1}$, while, at T>800 K, it was 7.7 × 10${}^{-6}$ K${}^{-1}$. The lower TEC at *T* < 800 K can be associated with two competing processes: (i) the redistribution of wrinkles and ripples in the graphene network during heating (this can result both in shrinkage and expansion of the CG) and (ii) thermal fluctuations of the atoms (the process is accompanied by the expansion of the CG). A similar explanation of the thermal expansion behavior for graphene is given in [39,76,94]. Moreover, at the temperature range 600–800 K, new covalent bonds started to appear in the graphene network, which also contributed to thermal expansion. The TEC of the CG stopped increasing after 800 K, since the appearance of new covalent bonds made the structure even more rigid.

The Ni/graphene composites of CG-8 at.% Ni, CG-16 at.% Ni, and CG-24 at.% Ni had a higher temperature expansion coefficient than that of crumpled graphene (see Figure 3b,c). This is associated with the presence of nickel nanoparticles, since Ni has a greater TEC than graphene (for bulk nickel α =13 × 10${}^{-6}$ K${}^{-1}$ at 300 K [95,96]). As can be seen, before 300 K, there was a difference between the TECs of CG-16 at.% Ni and CG-24 at.% Ni, while, after 300 K, the curves totally coincided. At the same time, the TEC of CG-8 at.% Ni before 300 K was much closer to the TEC of pure crumpled graphene, which means that, at low temperatures, thermal expansion was defined by the contribution of the graphene network, while, at high temperatures, the nickel nanoparticles affected the thermal expansion more. After *T* > 800 K, the TECs of all composites were close. Taking in accordance that the melting temperature of Ni nanoparticles is close to 1300 K [50,97] (and considerably dependent on the nanoparticle size), it can be concluded that the nanoparticles inside the crumpled graphene were melted or pre-melted at *T* > 800 K. At low temperatures, even a small difference in Ni content affects the TEC value of Ni/graphene composites. For all studied composites, the TECs were much lower than for pure Ni.

### 3.2. Thermal Conductivity

**Figure 4:**
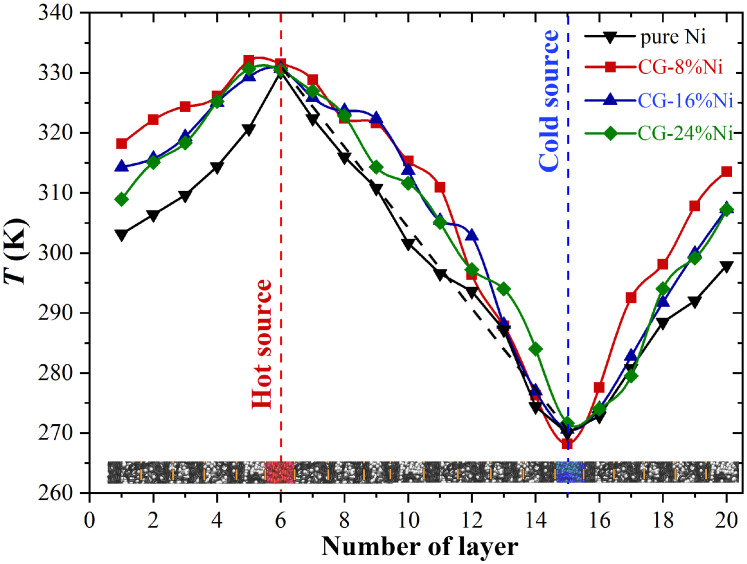
Temperature profiles for Ni/graphene composite and pure Ni after exposure at 300 K for 4 ps. Part of the structure is presented to show the temperature for each layer.

Figure 4 shows the temperature distribution over the simulation cell for pure Ni and Ni/graphene composites. The average temperatures of the “hot” and “cold” thermostat were 370 and 330 K, respectively. Part of the composite structure is also presented for convenience. The average temperature of each layer was calculated. Note that, for pure Ni, the temperature profile had a typical linear character (the black dashed curve between hot and cold sources in Figure 4). For metals, the heat transfer is more uniform than for Ni/graphene composites with strong covalent bonding and a complex crystal structure. The temperature profiles of the composites with nickel nanoparticles (Figure 4) were nonlinear.

**Figure 5:**
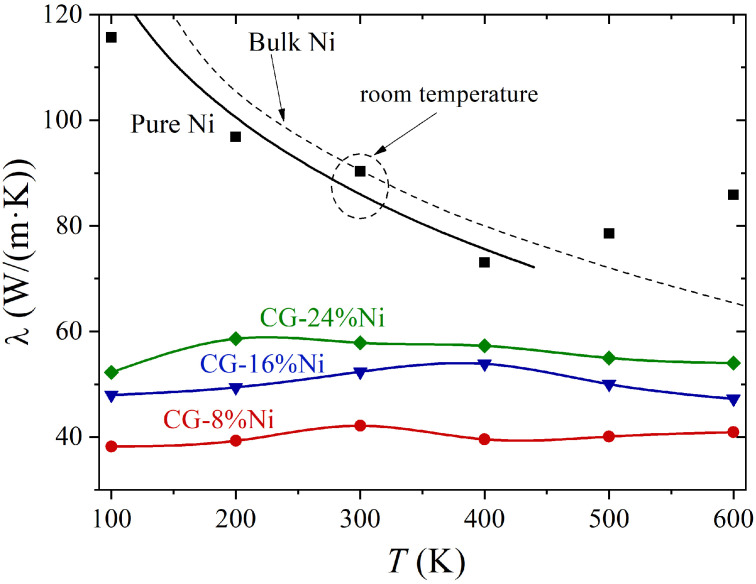
Thermal conductivity of pure Ni and Ni/graphene with different Ni content as a function of temperature. The dashed line shows the experimental data for bulk polycrystalline Ni [100]. The solid black line is the approximation of simulation data, shown by black squares.

Furthermore, the thermal conductivity was calculated for pure Ni and for all the studied Ni/graphene composites at different temperatures: *T* = 100, 200, 300, 400, 500, and 600 K. The thermal conductivity λ for the composites is shown in Figure 5. It can be seen that the thermal conductivity regarding each Ni/graphene composite depended on the Ni content: the higher Ni content, the higher the thermal conductivity at all temperatures. A decrease in thermal conductivity with the increase in graphene content was shown in [98] for Cu/graphene composites. However, the thermal conductivity for Ni/graphene composites slightly depends on temperature. The local deviations of λ for CG-16 at.% Ni and CG-24 at.% Ni were associated with the emerging new contact boundaries between Ni and graphene. Note that the obtained thermal conductivity at 100–200 K may have been underestimated due to the peculiarities of MD simulation [54,99], which only considers lattice thermal conductivity and does not take into account electronic thermal (phonon) conductivity. For metals and alloys, the effect of electronic thermal conductivity is not significant at *T* < 100 K. However, for Ni/graphene composites, electronic thermal conductivity can give a significant contribution to the thermal conductivity of the system, even at *T* > 100 K.

For pure Ni, the λ(T) curve was qualitatively similar to the experimental curve [100] characteristic of bulk polycrystalline Ni (black dashed curve in Figure 5). The results obtained by the simulation (black squares) could be approximated by the same line as the experimental one, but the values were lower. The observed difference is connected with differences in the sizes of the samples studied. In [90], it was shown that small-sized Ni nanoparticles had a lower thermal conductivity than bulk Ni. Furthermore, with an increase in the size of the nanoparticles, the thermal conductivity increased and approached the value of $\lambda_{bulk}$ = 91 W/(m K) (the thermal conductivity of bulk polycrystalline Ni at 300 K [100]). An increase in the thickness of Ni nanofilms also resulted in an increase in λ at 300 K to values that were characteristic of bulk Ni [101]. Note that, at room temperature, the thermal conductivity obtained in this work was equal to $\lambda_{bulk}$ (dotted circle in Figure 5). The thermal conductivity at 500 and 600 K was even higher than for bulk Ni. It can be explained by the melting or pre-melting of Ni samples in molecular dynamics. The size of the sample was small, which can result in a considerable decrease in melting temperature [50,97].

## 4. Discussion

**Figure 6:**
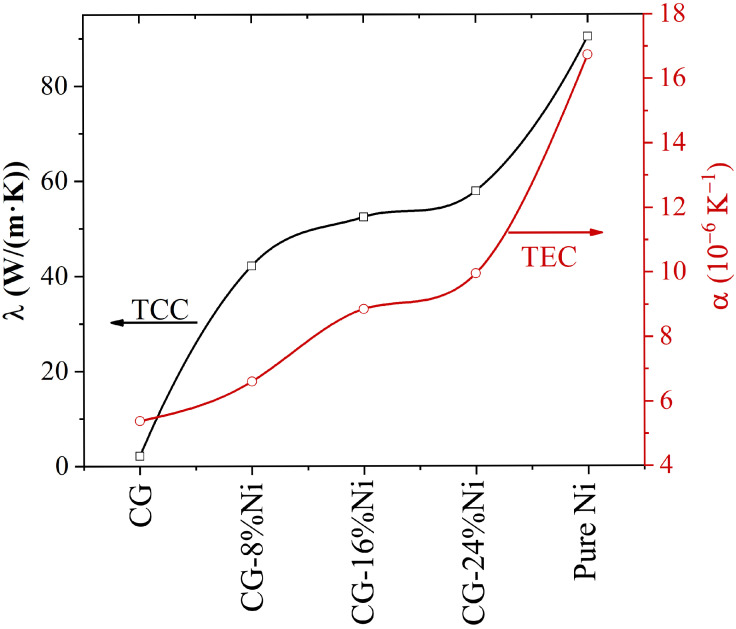
Coefficients of thermal conductivity (λ) and thermal expansion (α) of the studied structures at 300 K.

In Figure 6, the thermal conductivity (black curve) and TEC (red curve) for crumpled graphene, Ni/graphene composites, and pure Ni are presented for 300 K.

The thermal conductivity of single-layer graphene is known to be very high—from 3000 to 5000 W/(m K) at room temperature [24,25]. However, due to the considerable crumpling and wrinkling of CG, its thermal conductivity is considerably lower: 0.023 W/(m K) for N-doped graphene aerogel [102], 2.183 W/(m K) for graphene aerogel with octadecanoic acid [103], and 0.1 W/(m K) for graphene/carbon nanotube aerogels [104]. In [28], it was shown that temperature increases resulted in a sharp decrease in the thermal conductivity of graphene due to its wrinkling during heating. Furthermore, a decrease in the point defects density in the graphene also led to an increase in thermal conductivity [32,105].

The control of thermal properties through different internal and external factors is of great importance [106]. Furthermore, a variation in Ni content makes it possible to control the thermal expansion and thermal conductivity of composites. Therefore, an increase in Ni content in the composite leads to an increase in the thermal conductivity of the system (Figure 6). However, the TEC and thermal conductivity were very similar for CG-16 at.% Ni and CG-24 at.% Ni.

Several different factors can determine the thermal conductivity of Ni/graphene composites. For example, the small size of graphene flakes in the composite structure limits heat transition [107]. The introduction of Ni atoms transforms carbon atoms with $sp^{2}$ into $sp^{3}$ hybridization, which results in the destruction of the perfect π-electron-conjugated structure. The scattering of phonons (main heat delivery carriers) by defects and edges, or by $sp^{3}$ places, can significantly decrease the amount of heat. The appearance of new bonds between neighboring flakes also can reduce heat transfer.

The thermal conductivity of graphene is phonon thermal conductivity, since its electronic thermal conductivity is less than 1% of the total thermal conductivity of the system at room temperature [98]. In [26], the phonon thermal conductivity of graphene was studied, which was divided into six phonon branches—three acoustic and three optical. The LA phonon branch makes a greater contribution to thermal conductivity. However, for low temperatures (from 5 to 70 K), the ZA phonon branch has a greater contribution. It is rather difficult to carry out such an analysis of the separation of thermal scattering by phonon branches in the Ni/graphene composites under consideration, since they had a strongly deformed structure, which complicates such an analysis.

Table 1 shows summary data on the thermal and mechanical properties of the studied composites. Note that the mechanical properties of Ni/graphene composites with different Ni content were studied previously in [63]. The Ni content strongly affects both the thermal and mechanical properties of the Ni/graphene composites. The tensile strength and Young’s modulus decrease with an increase in the number of Ni atoms in the composite structure [48,63,108], while thermal conductivity simultaneously increases. Note that the obtained properties of the composites presented in Table 1 were higher than those of pure Ni or its alloys. These results make it possible to select such an atomic composition of Ni/graphene composite that will have the required properties.

## 5. Conclusions

The thermal properties of Ni/graphene composites, where crumpled graphene is a matrix and metal nanoparticles are fillers, were studied by molecular dynamics. It has been shown that the coefficients of thermal expansion and the thermal conductivity of composites strongly depend on the Ni content.

The thermal conductivities of the Ni/graphene composites were much lower than for pure Ni, but much higher than for crumpled graphene. The formation of a strongly deformed structure of crumpled graphene during its fabrication resulted in the formation of wrinkles and ripples, which significantly decreased the thermal conductivity in comparison with graphene. An increase in the amount of Ni atoms in the composite resulted in an increase in thermal conductivity due to the addition of an element with higher thermal conductivity. The formation of new contact boundaries between the nickel and carbon phases also had a strong effect on the thermal conductivity of the composite. The thermal conductivity of the Ni/graphene composite can be controlled by the changing Ni content.

The addition of nickel to the crumpled graphene led to an increase in thermal expansion. The TEC value of the CG-8 at.% Ni composite (containing a smaller number of nickel atoms in the structure) at a low temperature was closer to the TEC of crumpled graphene, while, at *T* > 300 K, it was considerably increased. The higher the Ni content in the composite, the higher the TEC value. At low temperatures, the thermal expansion was defined by the contribution of nickel nanoparticles, while, at high temperatures, the graphene network affected thermal expansion more.

The obtained results, in combination with previously published data on the improvement of the mechanical properties of Ni/graphene composites [48,63,108,109], give rise to their potential application. Such new metal/graphene composites have high mechanical properties and controllable thermal properties and can be used as alternative materials in different industries. For example, nowadays such composites can be used as chemical sensors, energy or hydrogen storage devices, for batteries, and for supercapacitors [3,4,40]. The development of the application of graphene-based composites toward energetic materials has been shown recently [4].

This work is just the start of the complex study of the physical and mechanical properties of graphene-based composites, which can be used for new electronics and protective coatings. In the future, we plan on considering the thermal properties of Me/graphene composites with metals with a higher thermal conductivity and TEC value than Ni nanoparticles, such as Cu and Al. This will expand the understanding of the thermal properties evolution of Me/graphene composites with metal fillers. This morphology is very new, and additional research is required for a better understanding of their future potential.

## Figures and Tables

**Table 1:** Thermal conductivity λ, TEC α, ultimate tensile strength $\sigma_{UTS}$, and Young’s modulus *E* [63]) of Ni/graphene composites at 300 K.

Composite	Thermal Properties		Mechanical Properties [63]	
	λ (W/(m K))	α (10${}^{-6}$ K${}^{-1}$)	*E* (GPa)	$\sigma_{\mathrm{UTS}}$ (GPa)
CG-8 at.% Ni	42.14	6.63	249	120
CG-16 at.% Ni	52.37	8.87	249	100
CG-24 at.% Ni	57.83	9.97	230	90

## Data Availability

Data are not available upon request due to restrictions, e.g., privacy or ethical.

## Abbreviations

The following abbreviations are used in this manuscript:

MD	Molecular Dynamics
TEC	Thermal Expansion Coefficient
TCC	Thermal Conductivity Coefficient
CG	Crumpled Graphene
